# Major depression with musical obsession treated with vortioxetine: a case report

**DOI:** 10.1186/s12991-021-00340-8

**Published:** 2021-03-09

**Authors:** Reiji Yoshimura, Naomichi Okamoto, Yuki Konishi, Atsuko Ikenouchi

**Affiliations:** Department of Psychiatry, University of Occupational and Environmental Health, Yahatanishiku, Kitakyushu, Fukuoka 8078555 Japan

**Keywords:** Major depression, Musical obsession, Vortioxetine

## Abstract

**Background:**

Musical obsession has been reported as the “stuck song syndrome” and can be accompanied by obsessive compulsive disorder (OCD). Musical obsession is the phenomenon where a particular set of known musical notes are perceived repeatedly. We present a case of major depression with musical obsession. In this case, vortioxetine improved both depressive symptoms and musical obsession.

**Case presentation:**

A female, 34-year-old, high school teacher presented with a depressed mood, anergia, difficulty in concentration, poor motivation, restlessness, anxiety, insomnia, and loss of appetite. She was diagnosed with major depression by her family physician and prescribed escitalopram (20 mg/day). Her depressive state partially responded to escitalopram. When she had been depressed, she also experienced musical obsessions as repetitive commercial tunes or instrumental notes inside her head that were not under conscious voluntary control and lasting several hours, causing a high level of distress in her daily life. After switching from escitalopram to vortioxetine (20 mg/day), her depressive symptoms and musical obsession symptoms were ameliorated.

**Conclusions:**

This case report endorses the utility of vortioxetine for major depression with musical obsession, and further studies should be conducted to establish the optimal treatment.

## Background

Musical obsession is the phenomenon of a particular set of known musical notes repeatedly being perceived. This phenomenon was first reported by Jacobs et al. in 1973 as a persistent or recurring paroxysmal auditory illusions evoking auditory perceptions, continuing for a certain period of time, after the initial stimulus has ended and was reported as a palinacousis by them [1]. Musical obsession was found to cause distress, anxiety or a depressive state [2].

We present a case of major depression with musical obsession. In this case, vortioxetine improved both depressive symptoms and musical obsession. To the best of our knowledge, this is the first report demonstrating the efficacy of vortioxetine in a case of major depression with musical obsession.

## Case presentation

A female, 34-year-old, high school teacher presented with a depressed mood, anergia, difficulty in concentration, poor motivation, restlessness, anxiety, insomnia, and appetite loss after her mother had died of cancer. She was diagnosed with major depression by her family physician and prescribed escitalopram (20 mg/day). Her depressive state partially responded to escitalopram, and her score on the Hamilton Rating Scale for Depression (HAMD) [3] decreased from 24 to 16 points 8 weeks after starting escitalopram. However, her depressive symptoms, including anergic symptoms, difficulty in concentration, and poor motivation continued. She consulted our university hospital after being suggested by her family physician. We found that when she had been depressed, she also experienced musical obsessions as repetitive commercial sounds or instrumental notes inside her head, that were not under conscious voluntary control and lasted several hours, causing a high level of distress in her daily life. She could not pay attention while reading books or talking to someone, and she also felt anxious. She was diagnosed with major depression with a musical obsession. Laboratory values were in the normal range for blood count, biochemistry, and endocrinological and ionogram analyses. MRI revealed no particular lesions in the brain. Her neurological examination, including electroencephalography, was also normal. She had no hearing loss. Her musical obsession did not respond to escitalopram. Thus, her medication was changed from escitalopram to vortioxetine (20 mg/day) because of her residual depressive symptoms and persistent musical obsession. Ten weeks after starting vortioxetine treatment, her HAMD scores decreased by 2 points. Her score on the Yale-Brown Obsessive Compulsive Scale (Y-BOCS) [4] before starting vortioxetine was 10 points. In short, she achieved remission. Her musical obsession gradually weakened and lasted only 10–20 min, 12 weeks after starting vortioxetine. Her score on the Y-BOCS decreased to 3 points. She said that she did not care about the musical obsession as before. Although she was still experiencing some musical obsession lasting a few minutes, once two or three weeks, she had no problems with her daily life, and did not see a relapse of the depressive episodes after maintenance therapy with 20 mg/day of vortioxetine.

## Discussion and conclusions

Musical obsession has been reported as the “stuck song syndrome or earworm” and can be accompanied by obsessive compulsive disorder (OCD) [2, 5].

In the present case, musical obsession occurred during the depressive episode. She had never experienced episodes of OCD in her lifetime. Findings from a European multicenter study demonstrated that concurrent OCD in MDD has a low prevalence rate [6]. Regarding the duration, symptomatic episodes vary and have been described as lasting from months to years, with a continuous or intermittent course [5]. It is difficult to distinguish between stuck song syndrome and OCD as the pathology of these disorders might be similar. Pfizer and Andrade, however, insisted that musical obsession is an additional unique characteristic of acute onset, marked severity, and occurs as an isolated symptom [7]. Regarding cases of certain distress and comorbid OCD symptoms, selective serotonin reuptake inhibitors (SSRIs) or clomipramine should be considered [5, 8]. Since she suffered from major depressive episodes and musical obsession, SSRIs were prescribed. Escitalopram partially ameliorated her depressive symptoms, but was not effective for musical obsession. When switching escitalopram to vortioxetine further improved her depressive symptoms, and she achieved remission of major depressive episodes ten weeks after starting vortioxetine. Moreover, her musical obsession subsequently relieved after her major depression had completely remitted. It remains unknown whether musical obsession is a part of the symptoms of major depression or is independent of major depression. Additionally, we cannot speculate the reason vortioxetine, but not escitalopram, reduced musical hallucination. Escitalopram and vortioxetine both belong to SSRLs. Vortioxetine has a broad action profile involving both serotonin (5HT) transporter and several 5HT receptors, including 5-HT_3A_, 5-HT_7_, and 5-HT_1D_ receptor antagonists, 5-HT_1B_ partial agonist, and 5-HT_1A_ agonist [9, 10]. Especially, we assumed antagonistic effect of vortioxetine on 5-HT_7_ might be related to the improvement of her musical obsession. Actually, it has been reported that inactivation and blockade of the 5-HT_7_ receptor reduced stereotypic behavior indicating that the 5-HT_7_ receptor might be of relevance as a target for the treatment of obsessive–compulsive disorder [11]. This pharmacological profile of vortioxetine might be related to its efficacy, although precise mechanisms must be elucidated. This case report endorses the utility of vortioxetine for major depression with musical obsession. However, further studies are needed to establish the optimal treatment.

## Data Availability

Not applicable.
